# Unveiling the Role of Tryptophan 2,3-Dioxygenase in the Angiogenic Process

**DOI:** 10.3390/ph17050558

**Published:** 2024-04-27

**Authors:** Marta Cecchi, Cecilia Anceschi, Angela Silvano, Maria Luisa Coniglio, Aurora Chinnici, Lucia Magnelli, Andrea Lapucci, Anna Laurenzana, Astrid Parenti

**Affiliations:** 1 Department of Neuroscience, Psychology, Drug Research and Child Health, (NEUROFARBA) Pharmacology and Toxicology Section, University of Florence, 50139 Florence, Italy; marta.cecchi@unifi.it (M.C.); aurora.chinnici@unifi.it (A.C.); 2 Department of Experimental and Clinical Biomedical Sciences “Mario Serio”, University of Florence, 50121 Florence, Italy; cecilia.anceschi@unifi.it (C.A.); lucia.magnelli@unifi.it (L.M.); anna.laurenzana@unifi.it (A.L.); 3 Department of Health Sciences, Division of Obstetrics and Gynecology, Careggi Hospital, University of Florence, 50134 Florence, Italy; angela.silvano@unifi.it; 4 Centre of Excellence, Division of Pediatric Oncology/Hematology, Meyer Children’s Hospital IRCCS, 50139 Florence, Italy; marialuisa.coniglio@meyer.it; 5 Department of Health Sciences, Clinical Pharmacology and Oncology Section, University of Florence, V. le G. Pieraccini, 6, 50139 Florence, Italy

**Keywords:** tryptophan 2,3-dioxygenase, angiogenesis, melanoma, kynurenine pathway, endothelial progenitor cells, endothelial cells, metalloproteinases

## Abstract

Background: Indoleamine 2,3-dioxygenase (IDO1) and tryptophan-2,3-dioxygenase (TDO) are the two principals enzymes involved in the catabolization of tryptophan (Trp) into kynurenine (Kyn). Despite their well-established role in the immune escape, their involvement in angiogenesis remains uncertain. We aimed to characterize TDO and IDO1 in human umbilical venular endothelial cells (HUVECs) and human endothelial colony-forming cells (ECFCs). Methods: qRT-PCR and immunofluorescence were used for TDO and IDO1 expression while their activity was measured using ELISA assays. Cell proliferation was examined via MTT tests and in in vitro angiogenesis by capillary morphogenesis. Results: HUVECs and ECFCs expressed TDO and IDO1. Treatment with the selective TDO inhibitor 680C91 significantly impaired HUVEC proliferation and 3D-tube formation in response to VEGF-A, while IDO1 inhibition showed no effect. VEGF-induced mTor phosphorylation and Kyn production were hindered by 680C91. ECFC morphogenesis was also inhibited by 680C91. Co-culturing HUVECs with A375 induced TDO up-regulation in both cell types, whose inhibition reduced MMP9 activity and prevented c-Myc and E2f1 upregulation. Conclusions: HUVECs and ECFCs express the key enzymes of the kynurenine pathway. Significantly, TDO emerges as a pivotal player in in vitro proliferation and capillary morphogenesis, suggesting a potential pathophysiological role in angiogenesis beyond its well-known immunomodulatory effects.

## 1. Introduction

Tryptophan (Trp) degradation and the consequent kynurenine (Kyn) production in the so-called kynurenine pathway is a mechanism exploited by different types of cancer to escape the immune system [1]. Three distinct players are involved in this catabolic pathway: indoleamine 2,3-dioxygenase (IDO1 and 2) and tryptophan 2,3-dioxygenase (TDO), although IDO2’s contribution to Trp metabolism appears to be less significant compared to the other two enzymes. Together, IDO1 and TDO catalyze the first and rate-limiting step of Trp oxidation yielding Kyn. Kyn indeed binds the aryl hydrocarbon receptor (AhR) which upregulates the expression of genes involved in immune suppression [2] and in melanoma progression. IDO1’s role in cancer biology has been extensively proven. However, studies on IDO1 expression in primary human melanoma are incomplete and conflicting. Moreover, the benefit of its inhibition in melanoma remains uncertain, highlighting the importance of considering TDO [3]. Until a few years ago, the characterization of TDO expression and its involvement in different kind of tumors were not taken into consideration. Recent reports have highlighted TDO’s involvement in the progression of some tumors [4,5]. We previously demonstrated that TDO is expressed by two human melanoma cell lines, namely A375 and SK-Mel-28 and regulates their phenotype and function [6,7]. Melanoma growth and metastasis depend on angiogenesis, the process of new capillary formation starting from pre-existing vessels [8]. The development of a rich vascular network is necessary to sustain melanoma cells, especially during the vertical growth phase, which is the most aggressive stage in tumor progression. This phase requires a lot of nutrients due to rapid cell proliferation [9]. Moreover, endothelial cells are leading contributors to the creation of the tumor microenvironment, thus favoring the spread of cancer cells. Another process that contributes to melanoma tumor vascularization is vasculogenesis, characterized by the creation of de novo blood vessels starting from endothelial progenitors [10]. Among endothelial progenitors, circulating progenitors of hematopoietic origin stimulate angiogenesis in a paracrine way but do not incorporate into blood vessels. Conversely, endothelial colony-forming cells (ECFCs), which are not of hematopoietic origin, have the ability to proliferate and form de novo blood vessels and colonies [11,12]. Studies demonstrated that ECFCs contribute to vascular network formation facilitated by cytokines and angiogenic factors released by melanoma cells and by melanoma-derived exosomes [13]. Furthermore, in vitro studies indicate that ECFCs can enhance the invasiveness of melanoma cells by up-regulating the urokinase plasminogen activator surface receptor (uPAR) [14]. Among pathways that are involved in melanoma-driven angiogenesis, there is no information on the kynurenine pathway. Moreover, it is unclear whether the endothelial kynurenine pathway contributes to the angiogenesis and vasculogenesis process. It has been reported that IDO1 may have a pro-angiogenic role in many cancer types [15] and, according to Zhang et al. [16], high IDO1 levels activate the IL-6/STAT3/VEGF-A pathway in bladder cancer cells, which contributes to the activation of the co-cultured endothelium. IDO1 expression was demonstrated to positively correlate with CD105^+^ endothelium in breast cancer and with a worse prognosis [17]. However, the information about the expression and function of TDO in endothelial cells is very limited. Recently, TDO expression was reported in non-tumoral cells, identified as pericytes in some tumors, suggesting its involvement in angiogenesis [18]. We demonstrated that human venular endothelial cells express TDO and its inhibition reduces their proliferation, indicating that TDO may be critical in angiogenesis [19]. However, its expression and function in endothelial cells and endothelial precursors are still doubtful. We aimed to better characterize IDO1 and TDO function in human endothelial cells and ECFCs by studying their in vitro function in the presence of selective inhibitors of IDO1 and TDO, i.e., epacadostat and 680C91, respectively. Additionally, a possible involvement of the kynurenine pathway in the melanoma–endothelial relationship was investigated.

## 2. Results

### 2.1. TDO and IDO1 Expression in HUVECs and ECFCs

**Figure 1:**
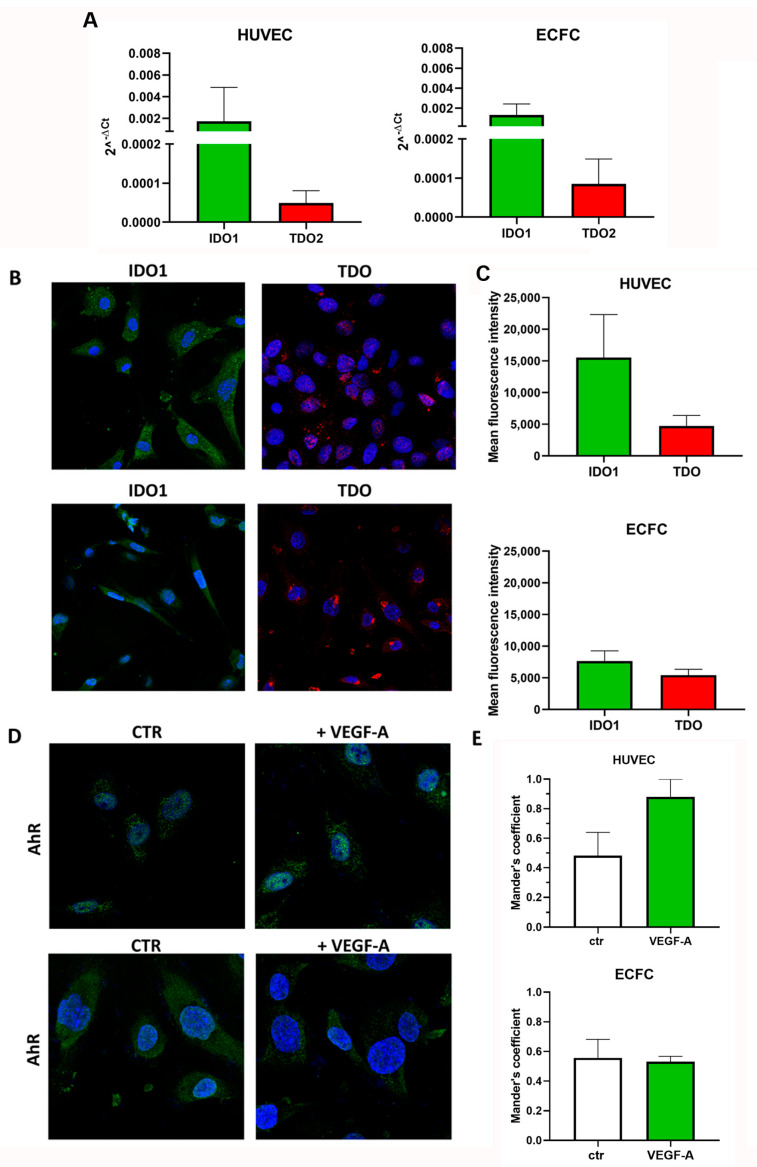
TDO, IDO1 and AhR expression in HUVEC and ECFC. (**A**) Real-time PCR analysis (mean ± SEM, *n* = 3). (**B**,**C**) Immunofluorescence, mean ± SEM, *n* = 3 and representative photomicrographs at 40× magnification for TDO (red) and IDO1 (green). (**D**,**E**) AhR (green) localization in the nucleus following the pro-angiogenic stimulus VEGF-A. Histograms show AhR fluorescence nuclear localization (AhR/DAPI) by Mander’s coefficient (M1) using Image J 1.54d software.

We previously demonstrated TDO mRNA (TDO2) expression levels in HUVECs [19], while no data are available in ECFCs. We then evaluated TDO2 expression in ECFCs compared to HUVEC levels; moreover, we studied IDO1 expression in both cell types. TDO2 was expressed by the two cell populations without any significant differences (Figure 1A,C); IDO1 mRNA was also detected in both cells, and its level was higher than that of TDO2 (Figure 1). Immunofluorescence confirmed TDO and IDO1 protein expression in both cells, with IDO1 being less expressed in ECFCs compared to HUVECs (Figure 1B,C).

Sanger sequencing on ECFCs cDNA obtained by reverse transcription was performed using primers spanning exon 6 and exon 7 of TDO2 cDNA and showed the presence of a complete amplified TDO2 region in RNA transcript (Figure S1). Consistently, Sanger sequencing on cDNA after real-time PCR confirmed TDO2 expression in HUVECs. 

Since Kyn, the product of tryptophan (Trp) catabolism via TDO and IDO1, binds and activates the aryl hydrocarbon receptor (AhR), the presence of an active kynurenine pathway was assessed measuring the protein level expression of AhR. Given that AhR is a transcriptional factor that translocates into the nucleus upon activation, we assessed its intracellular localization in response to a pro-angiogenic stimulus, specifically VEGF-A. In HUVECs, VEGF-A led to a moderate increase in AhR translocation into the nucleus, (Figure 1D,E). Intriguingly, this effect was not observed in ECFCs.

The ELISA assay for Kyn measurement confirmed the presence of an active kynurenine pathway in HUVECs, which was significantly stimulated by VEGF-A, as suggested by immunofluorescence analysis of AhR. Unstimulated cells, indeed, release 250.5 ng/mL Kyn, which increased up to 363.0 ng/mL following a 10 min stimulation with VEGF-A. Epacadostat, a selective IDO1 inhibitor, decreased VEGF-induced Kyn production to 122.31 ng/mL, while the TDO-selective inhibitor 680C91 completely prevented Kyn production (0.98 ng/mL). VEGF-A slightly increased Kyn production in ECFCs (168 ng/mL compared to unstimulated cells (150 ng/mL), which were not significantly impaired by either inhibitor. The levels were 155 ng/mL with 680C91 and 170 ng/mL with epacadostat.

### 2.2. Effect of TDO and IDO1 Inhibition on In Vitro Capillary-like Structures

**Figure 2:**
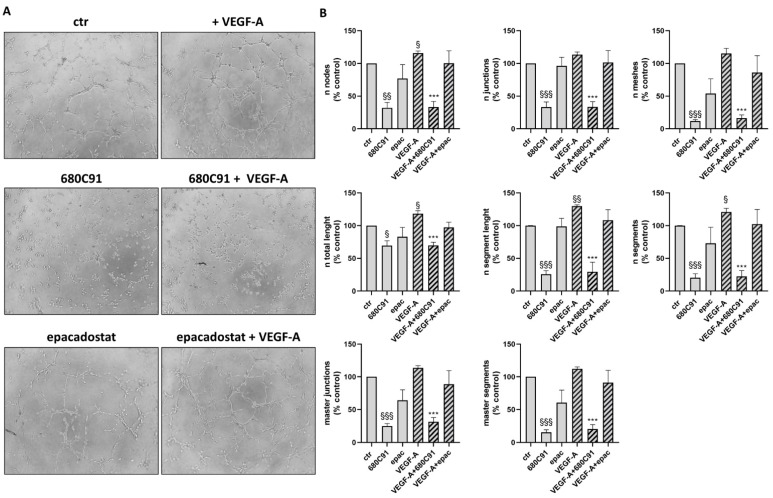
Effect of TDO and IDO1 inhibitors on in vitro angiogenesis. Angiogenesis was measured by capillary morphogenesis at 6 h in untreated HUVECs (ctr) and cells stimulated with 20 ng/mL VEGF-A in the absence or presence of 680C91 and epacadostat. (**A**) Representative pictures, 10× magnification. (**B**) Mean ± SEM, *n* = 3. ^§^ *p* < 0.05; ^§§^ *p* < 0.04; ^§§§^ *p* < 0.001 vs. untreated (ctr). *** *p* < 0.001 vs. VEGF-A.

The addition of VEGF-A to HUVECs significantly stimulated capillary morphogenesis, as expected (Figure 2). The quantification of the formed capillary network has been evaluated using different parameters, such as the number of master junctions, nodes, segments and meshes formed or the number of total segments, which is the most important parameter. Interestingly, 680C91 significantly impaired spontaneous network formation (Figure 2) and the addition of VEGF-A failed to mitigate this inhibitory effect. Conversely, the IDO1 inhibitor, epacadostat, demonstrated a modest impairment in spontaneous pseudocapillary formation. Notably, this effect did not counteract the angiogenic effect of VEGF-A, thereby highlighting a nuanced interplay between the two inhibitors and emphasizing the intricate regulatory mechanisms governing angiogenic processes in our experimental context.

**Figure 3:**
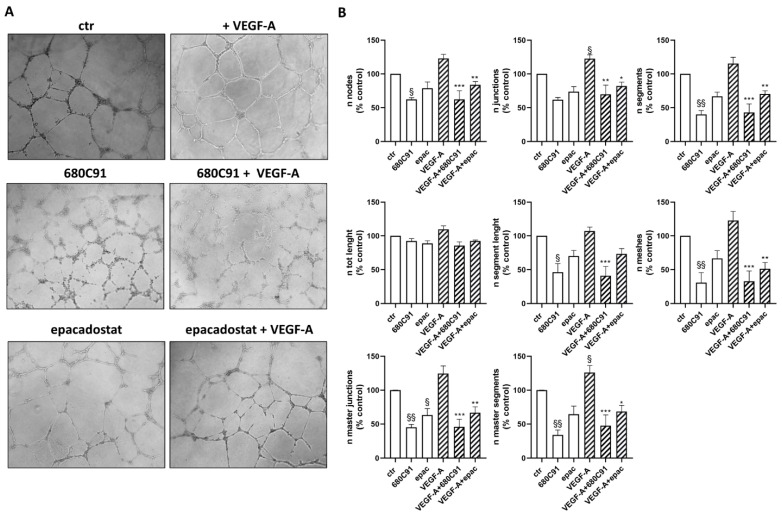
Effect of TDO and IDO1 inhibitors on in vitro vasculogenesis. Vasculogenesis was measured by capillary morphogenesis at 24 h in untreated ECFCs (ctr) and cells stimulated with 20 ng/mL VEGF-A in the absence or presence of 680C91 and epacadostat. (**A**) Representative pictures. 10× magnification. (**B**) Mean ± SEM, *n* = 3; ^§^ *p* < 0.05; ^§§^ *p* < 0.01 vs. untreated (ctr). * *p* < 0.05; ** *p* < 0.01; *** *p* < 0.001 vs. VEGF-A.

ECFCs have been reported to be involved in angiogenesis together with mature endothelial cells. When seeded on Matrigel, they formed capillary-like structures within 24 h [20]. The inhibition of TDO markedly impaired the ability of ECFCs to form tubes. Although VEGF induced a modest increase in capillary-like structures, the effect was less pronounced compared to HUVECs. Crucially, it was unable to counteract the inhibition induced by 680C91, mirroring the observed outcomes in HUVECs (Figure 3). On the other hand, inhibiting IDO1 did not disrupt spontaneous pseudo-capillary formation and, interestingly, partially hindered the induction by VEGF. These findings underscore the intricacies and distinct responses in capillary formation in this context, highlighting the unique interplay between VEGF, 680C91 and IDO1 in modulating angiogenic processes.

Considering these results, and the previous ones obtained by ELISA assay, demonstrating a scarce activation of the kynurenine pathway in ECFCs, we conducted a more in-depth exploration into the role of TDO in HUVECs’ function, particularly under stimulation with angiogenic factors, including VEGF-A.

### 2.3. TDO but Not IDO1 Is Involved in HUVEC Proliferation

**Figure 4:**
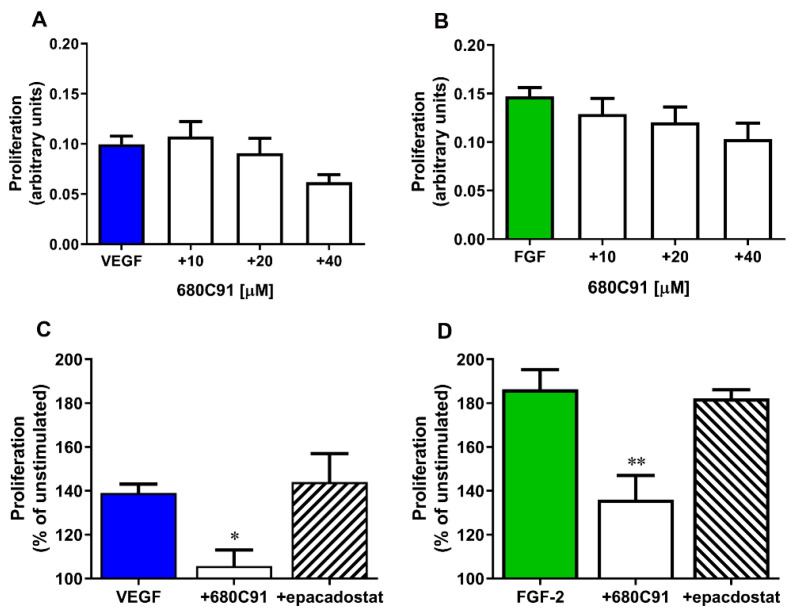
Effect of the TDO inhibitor 680C31 and IDO1 inhibitor epacadostat on HUVEC proliferation. Cell proliferation was evaluated in response to (**A**,**C**) VEGF-A and (**B**,**D**) FGF-2 in the presence of increasing concentrations of 680C91 (**A**,**B**). (**C**,**D**) Effects of the highest concentration of 680C91 (40 µM) and epacadostat (1 µM) on VEGF-A- and FGF-2-induced HUVEC growth. Mean ± SEM, *n* = 5; * *p* < 0.05 vs. VEGF-A alone; ** *p* < 0.01 vs. FGF-2 alone.

We previously demonstrated that TDO is involved in HUVEC proliferation. Specifically, 680C91, the TDO selective inhibitor, significantly inhibited HUVEC proliferation in response to optimal growth conditions (10% fetal bovine serum (FBS)), in a concentration-dependent manner, with maximal effects observed at 40 µM 680C91 [19]. We investigated the possible role of TDO and IDO1 on the proliferative effect of VEGF-A on HUVECs and we compared it with the effect of another important angiogenic factor, FGF-2. Cell treatment with 680C91 significantly impaired VEGF-A-stimulated cell proliferation in a dose–response manner, with a maximal effect at the highest concentration (40 µM) that was able to inhibit cell growth by 79.3 ± 28% (Figure 4A,C). Cell proliferation in response to FGF-2 was also affected by the TDO inhibitor, in a concentration-dependent manner. The highest concentration of 680C91 impaired FGF-2-induced HUVEC growth by 60.73 ± 12% (Figure 4B,D). Conversely, inhibition of IDO1 did not modify VEGF-A nor FGF-2 effects (Figure 4C,D). Both inhibitors did not affect basal proliferation.

### 2.4. Endothelial Signaling Linked to TDO Activation

To investigate the mechanistic processes behind TDO’s involvement in VEGF-induced proliferation of HUVECs, we studied the activation of mTOR, a pathway known to be related to angiogenesis in many cancers. Cells were quickly stimulated with VEGF-A in the presence or absence of the TDO selective inhibitor and the activation of, mTOR was evaluated based on the quantification of their phosphorylated forms.

**Figure 5:**
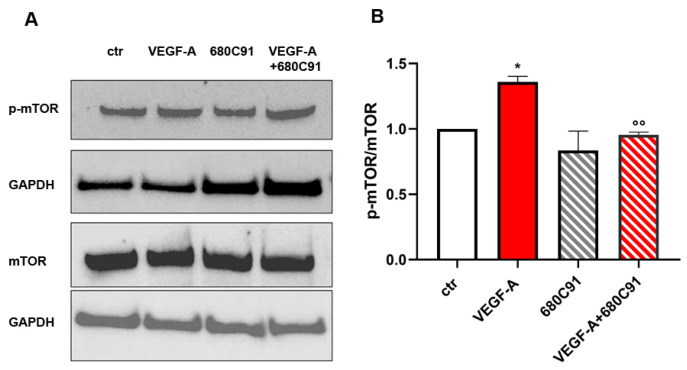
m-TOR activation in response to VEGF-A stimulation in the presence of TDO selective inhibitor. HUVECs were untreated (ctr) or treated with VEGF-A (20 ng/mL) for 10′ ± 680C91 (40 µM). mTOR activation was evaluated following a densitometry analysis of Western blots by means of p-mTOR/mTOR. (**A**) Representative experiment; (**B**) Mean ± SEM, *n* = 3. * *p* < 0.05 vs. ctr, °° *p* < 0.01 vs. VEGF-A.

MTOR resulted in being activated in response to VEGF-A as expected, and interestingly, this effect was significantly prevented by 680C91 treatment (Figure 5).

### 2.5. Role of TDO on A375-HUVEC Communication

**Figure 6:**
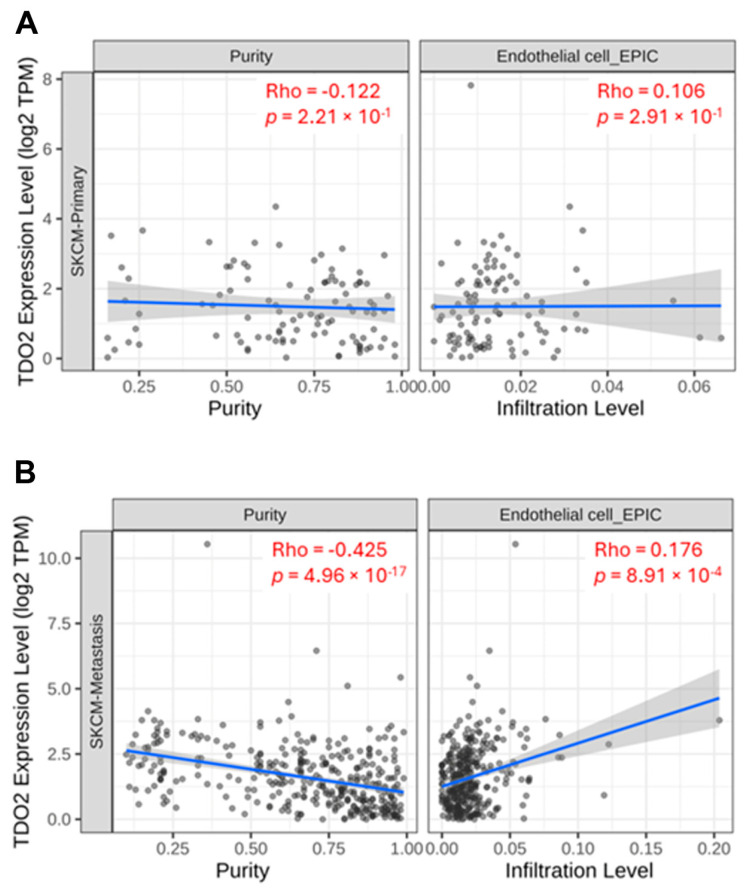
TIMER 2.0 correlation analysis between TDO expression and endothelial cell infiltration in primary (**A**) and metastatic (**B**) melanoma. Spearman’s *ρ* (Rho): no significant correlation *p* > 0.05 (**A**), positive significant correlation *p* < 0.05, *ρ* > 0 (**B**).

The angiogenic process is of primary importance for melanoma progression and metastasis and is based on the production and release of molecules with a pro-angiogenic effect in the tumor microenvironment. Melanoma–endothelial cell communication has been reported to influence molecular signals mediating tumor growth and progression. With the online tool TIMER2.0 (http://timer.cistrome.org/ accessed on 12 January 2024), which is a server able to perform associations between immune infiltration and different tumor characteristics using data from The Cancer Genome Atlas (TCGA) [21], we performed an analysis which indicated that TDO expression positively correlates with the infiltration of endothelial cells in metastatic but not in primary melanoma (Figure 6). 

**Figure 7:**
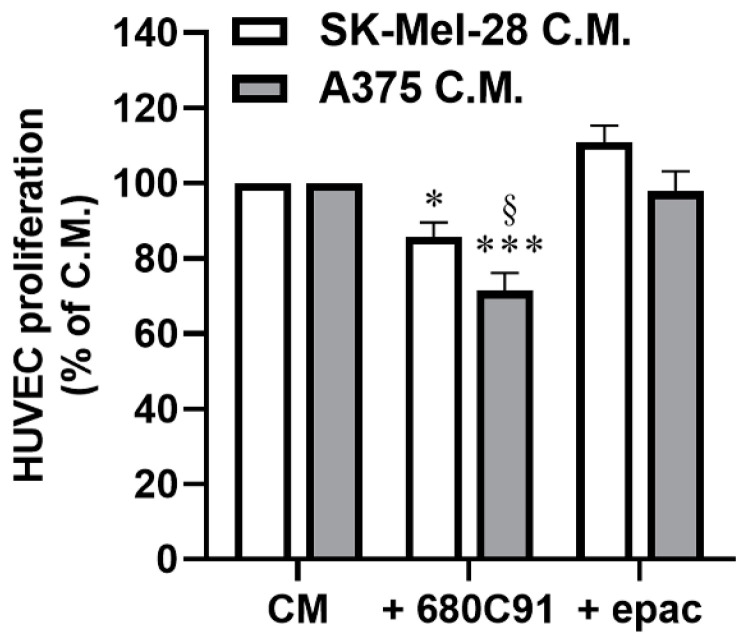
HUVEC proliferation in response to A375 or SK-Mel-28 conditioned media (C.M.). Melanoma cells were treated with 680C91 (40 µM) or epacadostat (1 µM) for 24 h, and then media were recovered, clarified and added to HUVECs. After 2 days, HUVEC proliferation was assessed. Mean ± SEM, *n* = 4. * *p* < 0.05, *** *p* < 0.001 vs. untreated C.M. ^§^ *p* < 0.05 vs. SK-Mel-28 C.M.

The interplay of endothelial and cancer cells in the tumor microenvironment is well known. Since we already demonstrated the key role of TDO in different human melanoma cell lines and based on the observed positive correlation between TDO expression and endothelial cell infiltrations, we then investigated the possible role of TDO in endothelial cells’ behavior in the presence of malignant human melanoma cells SK-Mel-28 and A375, which have been reported to exhibit different levels of invasiveness [22]. We studied endothelial cell proliferation in response to differently treated melanoma cells. A375 and SK-Mel-28 were grown in a medium +1% FBS in the absence or presence of TDO and IDO1 inhibitors. Then, the media were clarified and added to HUVECs, and their proliferation was assessed. As shown in Figure 7, pretreatment of A375 with the TDO inhibitor impaired the proliferation of endothelial cells. The inhibition of endothelial growth was more pronounced when exposed to media recovered from 680C91-treated A375 cells compared to media derived from 680C91-treated SK-Mel-28. Conversely, the inhibition of IDO1 in both melanoma cells had no discernible impact on HUVEC growth (Figure 7). Based on these results, A375 cells were then selected for further experiments focusing on investigating the role of TDO in the intricate communication between melanoma and endothelial cells.

**Figure 8:**
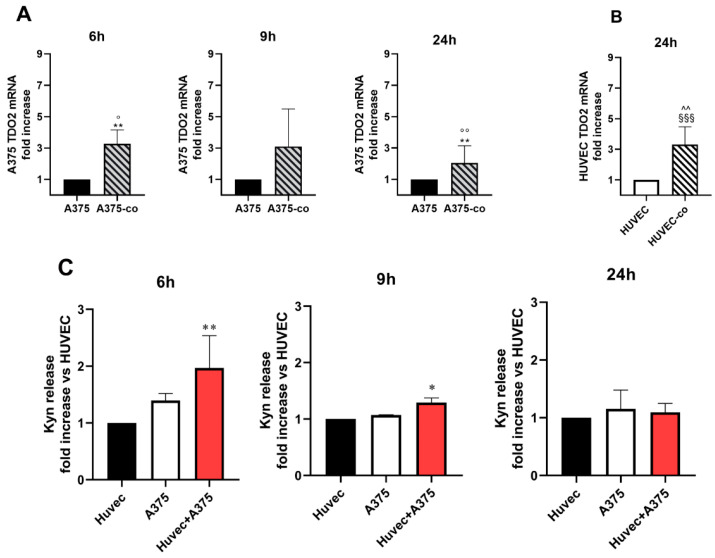
(**A**,**B**) RT-PCR for TDO2 expression and C) Kyn release in A375 HUVECs and co-cultures. (**A**) RT-PCR in A375 alone and in A375 co-cultured with HUVECs (A375-co). Time course. Mean ± SEM, *n* = 5. ** *p* < 0.01 vs. A375 alone; ° *p* < 0.05, °° *p* < 0.01 vs. HUVEC alone; (**B**) RT-PCR in HUVECs alone and in HUVECs co-cultured with A375 (HUVEC-co). Mean ± SEM, *n* = 3. ^§§§^ *p* < 0.001 vs. HUVECs alone; ^^ *p* < 0.01 vs. A375 alone. (**C**) ELISA assay for Kyn production in HUVECs, A375 and in the co-cultures (Huvec + A375) for 6, 9 and 24 h. Mean ± SEM. *n* = 5. * *p* < 0.05, ** *p* < 0.01 vs. HUVECs alone. The Kruskal–Wallis test and Dunn’s post hoc test were performed.

The mutual influence between human melanoma cells and endothelial cells was investigated by means of co-cultures by assessing TDO2 and IDO1 expression in A375 and HUVECs. The co-culture setup involved the juxtaposition of the two cell types, sharing the culture medium but physically separated by the membrane of the insert. Interestingly, A375 co-cultured with endothelial cells expressed higher TDO2 mRNA within 6 h stimulation. These levels were significantly higher than those expressed by A375 and HUVECs alone (Figure 8A). A 24 h co-culture also induced a significant up-regulation of TDO2 in HUVECs compared to A375 and HUVECs alone (Figure 8B). Conversely, IDO1 mRNA expression did not change in the co-culture (Figure S2).

To evaluate whether TDO2 upregulation correlates with its enzymatic activity, the release of Kyn was measured by ELISA assay. A significant increase in Kyn production was measured following 6 h and 9 h of co-culture compared to that released by HUVECs alone, and then Kyn release became comparable between co-cultured and parental cells (Figure 8C).

**Figure 9:**
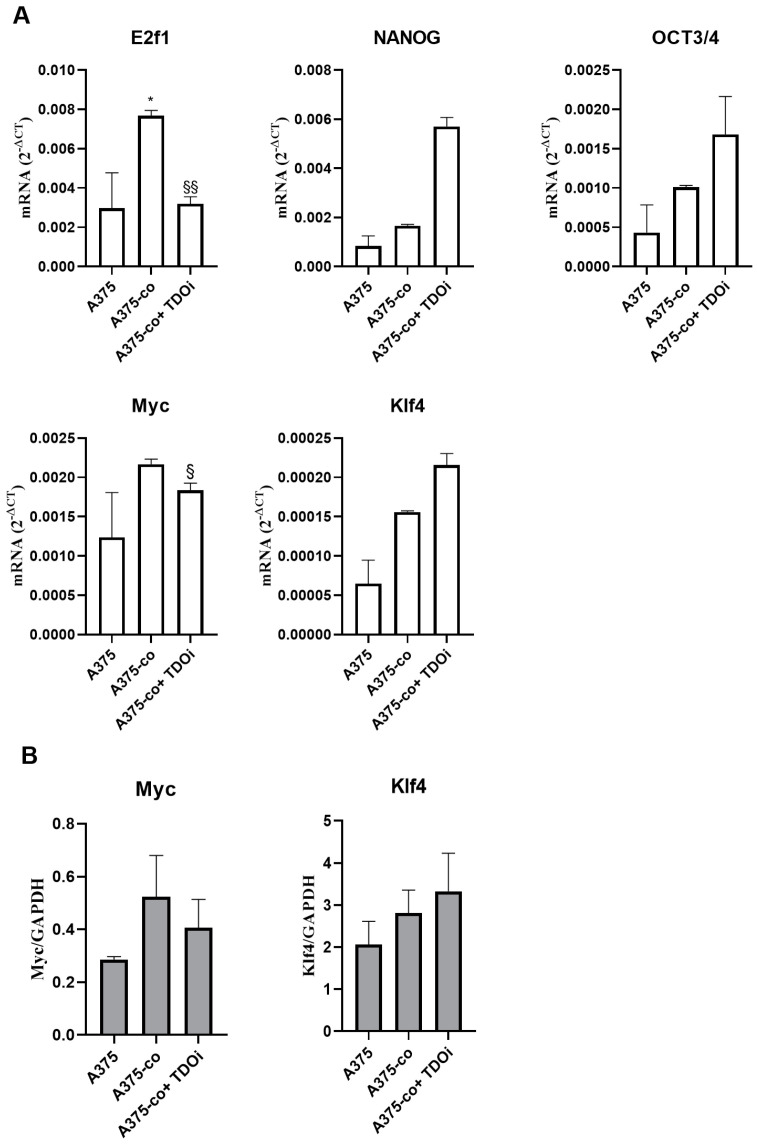
(**A**) Relative mRNA expression levels of stemness markers in A375 and in A375 co-cultured for 24 h with HUVECs in the presence or absence of the TDO inhibitor. After 24 h, total RNA was extracted to perform real-time PCR. Data are shown as Mean ± SEM, *n* = 3. * *p* < 0.05 vs. A375 alone, ^§^ *p* < 0.05, ^§§^ *p* < 0.01 vs. A375 co-cultured. (**B**) Protein expression for Myc and Klf4 in A375 and in A375 co-cultured (A375-co) for 24 h with HUVECs in the presence of the TDO inhibitor 680C91. Mean ± SEM, *n* = 3.

Since the influence of the tumor microenvironment is a crucial element in the transformation of differentiated cells into undifferentiated ones, we evaluated the expression levels of some of the pluripotency stem cell markers in A375 melanoma cells co-cultured with endothelial cells. Moreover, we studied the possible role of TDO in their regulation using the TDO selective inhibitor 680C91. As shown in Figure 9, the co-culture of the A375 cell line with the endothelial line shows an up-regulation of Myc and E2F1 genes; these effects were significantly prevented in the presence of the TDO inhibitor 680C91. Of note, KLF4, Nanog and OCT3/4 were up-regulated in the TDO inhibitor-treated group. The protein expressions of Myc and KLF4 were in line with that of mRNA. Specifically, Myc expression increased in A375 co-cultured with HUVECs, and this increase was partially prevented by the TDO inhibitor 680C91. On the other hand, KLF4 expression was elevated in A375 co-cultured with HUVECs and was further increased in the presence of 680C91. While this effect was unexpected, it could be explained by the critical balance between pathways that could promote or inhibit the progression of malignancies.

### 2.6. MMP Release in the Co-Cultures

**Figure 10:**
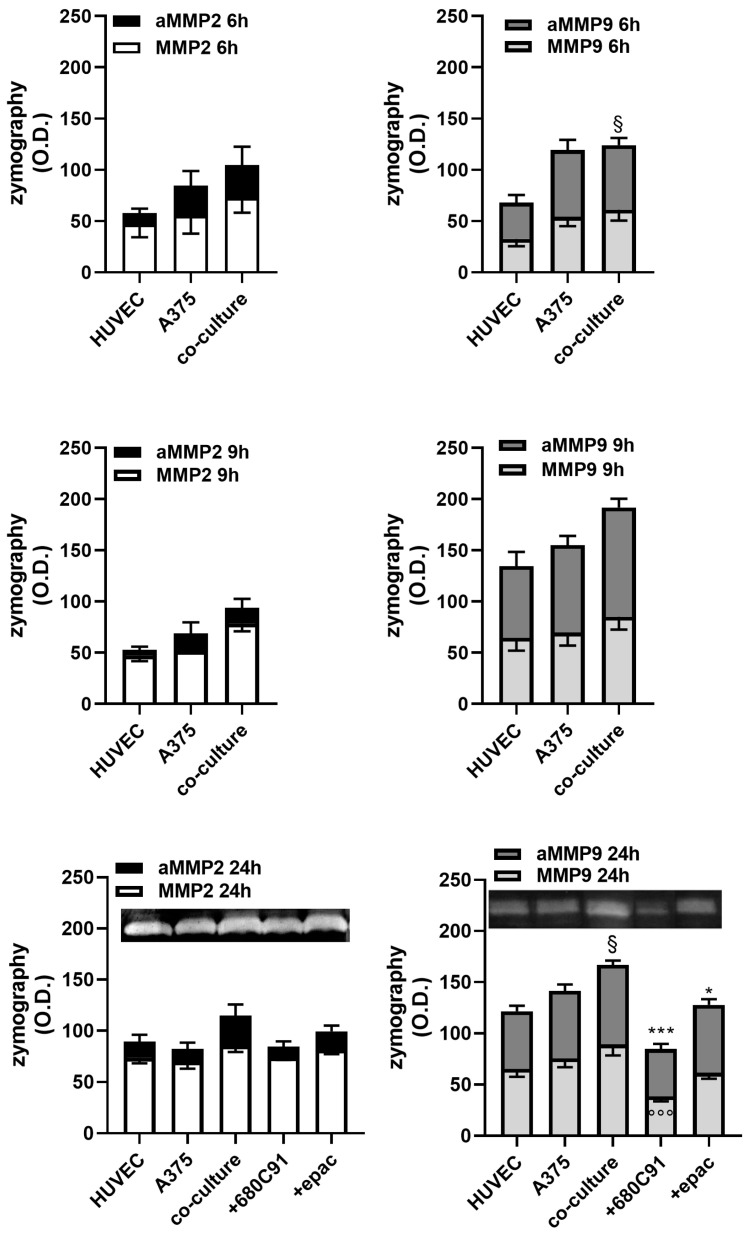
Gelatin zymography for MMP2 and MMP9 activity in A375, HUVECs and in A375 co-cultured with HUVECs (co-culture) for 6–24 h. In some experiments, A375 was pretreated overnight with 680C91 or epacadostat and then co-cultures were set up. Densitometric analysis shows the effect of co-culture on latent and activated (aMMP2) MMP2 and on latent and activated (aMMP9) MMP9 at 24 h representative zymograms. Mean ± SEM of 5 experiments. ^§^ *p* < 0.05 vs. activated MMP9 of HUVECs; * *p* < 0.05, *** *p* < 0.001 vs. activated MMP9 of co-culture; °°° *p* < 0.001 vs. latent MMP9 of co-culture.

MMP-2 and MMP-9 are involved in angiogenesis and melanoma cell invasiveness [23,24]. In our study, we examined the release of these metalloproteinases in A375 cells, HUVECs and A375 co-cultured with HUVECs over a 24 h period. Furthermore, we investigated the influence of TDO and IDO1 inhibitors on MMP-2 and MMP-9 released by these co-cultures. A375 melanoma cells were treated overnight with 40 µM 680C91 or 1 µM epacadostat, and then co-cultures with HUVECs were established. Gelatin zymography of A375 cell supernatants displayed constitutive release of the latent and activated forms of MMP-2 and MMP-9 (Figure 10). Their activities were higher than that of HUVECs. Time-course experiments showed an increase in MMP-2 and a significant production of both latent and activated forms of MMP-9 in the co-cultures with maximal activity following a 24 h co-culture, which were significantly impaired by TDO inhibition (Figure 10).

## 3. Discussion

Metastatic melanoma is the most aggressive and lethal form of skin cancer and it is characterized by rapid growth, high rates of late-stage recurrence and extensive metastasis [25,26]. Although there are plentiful clinical therapeutic options, the prognosis of advanced melanoma remains severe [27]. Angiogenesis and vasculogenesis are essential for the occurrence and development of melanoma because tumor cells need lots of nutrients and oxygen to sustain their vertical growth [9]. Among angiogenic factors released by melanoma cells, VEGF-A is overexpressed and associated with prognosis in melanoma patients [28]. Indeed, targeting angiogenesis has been considered a promising strategy in cancer therapy [29]. However, anti-angiogenic therapy effectiveness is often compromised by the emergence of drug resistance, particularly in patients with melanoma and other metastatic cancers [30,31]. The predominant strategies employed thus far for inhibiting the VEGF axis involve targeting either VEGF receptors (VEGFRs) or ligands using neutralizing antibodies or inhibiting receptor tyrosine kinase (RTK) enzymes. Despite encouraging outcomes observed in preclinical experiments, the clinical efficacy of anti-angiogenic monotherapies has been modest. This limited efficacy could be attributed to the development of primary or acquired resistance, which arises from growth factor redundancy and recruitment of various cell types and the activation of alternative mechanisms supporting tumor vascularization and growth such as vascular co-option and vasculogenic mimicry [30,32].

The role of the kynurenine pathway in tumor neovascularization is still unknown. Some data have been reported for IDO1, which was demonstrated to have a pro-angiogenic role in ovarian types [33] and to be involved in vasculogenic mimicry of lung cancer cells [15]. However, a direct effect of IDO1 inhibition on HUVECs has never been investigated. No information is available on TDO and tumor angiogenesis. In this paper, we demonstrate that human endothelial and endothelial precursors express both the enzymes of the kynurenine pathway and the downstream target AhR and that IDO1 is more expressed than TDO. This higher expression of IDO1 than TDO is not surprising, at least in HUVECs, as IDO1 is considered the main enzyme of the kynurenine pathway expressed in nearly all tissues, while TDO is almost localized in the liver, which is one of the reasons why IDO1 is in general studied more than TDO [1]. However, our data demonstrated that TDO is more involved in the regulation of endothelial cells in in vitro functions. Its inhibition, indeed, prevented pseudocapillary formation when HUVECs and ECFCs were seeded on Geltrex. It is crucial to acknowledge that the sensitivity of endothelial cell lines to VEGF-A is contingent upon the specific cell types, due to the differential expression of angiogenic markers including VEGFR2 receptors [34]. Furthermore, recent reports have highlighted the role of lipid rafts in stabilizing the VEGFRs, indicating that the modulation of these rafts can exert control over the sensitivity of endothelial cells to VEGF stimulation [20,35]. Notably, our observations reveal a distinct and time-dependent responsiveness of HUVECs and ECFCs to VEGF-A in the context of capillary structure formation. This divergence mirrors their inherent angiogenic potential, with HUVECs demonstrating the ability to form mature capillary structures within 6 h, while ECFCs achieve similar organization over a 24 h period. Furthermore, our research highlights the superior efficacy of TDO inhibition over IDO1 inhibition in disrupting spontaneous capillary formation. Significantly, TDO inhibition not only impedes spontaneous capillary formation but also effectively counters the proangiogenic effects induced by VEGF-A in both cell lines, albeit with differing magnitudes of impact. Of particular interest is the observation that the inhibitory effects of IDO1 inhibition in HUVECs can be completely overturned by VEGF-A, illustrating a unique responsiveness compared to ECFCs. In the case of ECFCs, we observe the persistence of IDO1 inhibition even in the presence of VEGF-A, possibly attributable to the reduced responsiveness of these cells to VEGF stimuli. These findings shed light on the intricate dynamics of angiogenesis regulation in different endothelial cell lines and highlight the varying impacts of TDO and IDO1 inhibition on capillary formation and response to VEGF-A.

TDO inhibition also impairs FGF-2- and VEGF-A-induced HUVEC proliferation. We previously reported that TDO inhibition affected the cell cycle of HUVECs without inducing cell apoptosis or cell death [19]. Among the intracellular pathways involved, we focused on the mTOR pathway, which plays a pivotal role in cell proliferation and angiogenesis. Experimental evidence demonstrates that suppressing the mTOR pathway effectively hinders VEGF-mediated angiogenesis and cell proliferation by attenuating VEGF-A production and secretion. Additionally, mTOR serves as a paramount metabolic regulator, functioning as a critical nutrient sensor that dictates cell growth versus autophagy decisions, particularly under conditions of amino acid deprivation. Recent findings have unveiled a functional loop between the Kyn and the mTOR pathway [36]. The authors described the role of IFNγ, a robust inducer of IDO1, in depleting tryptophan and simultaneously activating mTOR. Our data reveal a compelling correlation between the inhibition of the kynurenine pathway through 680C91 and the interference with VEGF-A-induced angiogenesis through the modulation of the mTOR pathway. Specifically, by targeting the kynurenine pathway, we observed a pronounced disruption in the angiogenic process orchestrated by VEGF-A.

These data underscore the intricate interplay between the mTOR pathway, angiogenesis, and cellular metabolism, shedding light on potential avenues for therapeutic interventions and advancing our understanding of cellular regulatory mechanisms. The possible involvement of TDO in melanoma cell–endothelial cell communication was investigated. We demonstrate that HUVECs’ proliferation in response to the conditioned media of melanoma cells depends in part on TDO function since it is inhibited by 680C91, while IDO1 inhibition with epacadostat did not modify the proliferative potential of the melanoma-conditioned medium. The influence of co-cultured cells on TDO expression was then investigated and we demonstrated a significant upregulation of TDO2 mRNA in both cells, i.e., A375 and HUVEC, suggesting a pathophysiological role. As a matter of fact, in the present paper, we demonstrate that TDO inhibition, but not IDO1 inhibition, significantly reduced MMP-9 activity of melanoma cells co-cultured with endothelial cells.

Following melanoma and endothelial cells’ co-culture, we also demonstrated an increased expression of the stem cell markers E2f1 and c-Myc in A375, and this up-regulation was prevented by TDO inhibition. Interestingly, OCT3/4, NANOG and Klf4 mRNAs were up-regulated by 680C91, suggesting a critical role of stem cell markers in regulating melanoma cell malignancies.

It is well known that cancer cells tend to mimic pluripotent stem cells’ behavior through the activation of pathways and pluripotency-associated transcriptional factors. In cancer stem cells, Myc, Nanog, Oct4, KLF4 and Sox2 are established to be the leading factors for stemness [37]. Other transcriptional factors, such as E2F1, are expressed by a variety of solid tumors and are involved in the regulation of cancer cells’ proliferation and self-renewal [38]. Overexpression of E2F1 has been demonstrated to be a negative prognostic factor for patient’s survival and, interestingly, its expression is higher in melanoma metastasis rather than in the primary tumor [39]. In fact, inhibition of E2F1 has been reported to decrease the metastatic potential of melanoma cells [39], pointing out the importance of E2F1 de-regulation not only in cancer cells’ proliferation but also in melanoma invasion [39].

There is increasing evidence describing KLF4 as a double effector in tumor development. While this stem cell marker can promote the progression of some malignancies, it is also known as a tumor suppressor. For example, it has been reported that KLF4 can promote cell differentiation, inhibition of cell cycles and also the activation of death pathways in neuroblastoma [40]. Moreover, the regulatory role of KLF4 has been described in a wide number of cancers, such as lung, breast, prostate, colorectal, brain cancer and more. Nevertheless, the implication of KLF4 in tumorigenesis is very intricate, probably due to its structure which retains both transcriptional activation and repression domains, and through regulation of its upstream and downstream signaling molecules [41]. While Nanog and OCT3/4 are mostly known for their pro-tumorigenic role, recently, a study performed in mice skin cells demonstrated that the tumor suppressor p53 can be activated by Nanog, leading to anti-tumorigenic effects [42]. Moreover, OCT3/4 also needs to be fully elucidated. OCT3/4 is part of the family of transcription factors holding the POU domain. The three main transcripts that are known are OCT3/4A, OCT3/4B and OCT3/4B1. It is reported that OCT3/4 has a role in the promotion of cervical cancer carcinogenesis and the development of malignant tumors [43].

Indeed, the upregulation of OCT3/4 in response to the selective TDO inhibitor is in line with our previous results obtained in A375 parental cells and fueled our interest in some reports where the less-known role of this stem marker was demonstrated. Shen et al. [44] studied Oct4 involvement in breast cancer metastasis and they interestingly found out that its overexpression could inhibit cell migration and the formation of lung metastasis in vivo, while its downregulation heightened the metastatic potential of breast cancer cells both in vitro and in vivo.

In conclusion, our data can support the hypothesis that endothelial cells possess a functional TDO which regulates their function. Therefore, TDO may contribute not only to tumor growth through an immune system evasion mechanism but also by influencing cells in the tumor microenvironment, such as endothelial cells, establishing crosstalk with them. Anti-angiogenic therapy is not as effective, and drug resistance always occurs in patients with melanoma [45]. It becomes of increased importance for future research to better characterize the involvement of the TDO pathway in the angiogenic process stimulated by melanoma cells. A more in-depth comprehension of the angiogenic process will be important to improve melanoma treatment and may promote the discovery of new targets to further increase the availability of melanoma therapies.

## 4. Materials and Methods 

### 4.1. Cell Culture

Human metastatic melanoma cell line A375 (ATCC, Manassas, VA, USA) was grown in high D-glucose DMEM, with 10% (*v*/*v*) heat-inactivated fetal bovine serum (Euroclone Milan, Italy), 100 U/mL penicillin, 100 μg/mL streptomycin and 2 mmol/L glutamine in a humidified atmosphere with 5% CO_2_ in air. The culture medium was changed every 2 days. HUVECs (ATCC, Manassas, VA, USA) were grown in Endothelial Basal medium, supplemented with 10% FBS (Euroclone, Milan, Italy), on gelatin-coated dishes. Endothelial Colony Forming Cells (ECFCs) were isolated from >50 mL human umbilical cord blood (UCB) of healthy newborns after maternal informed consent as previously described [20,46] The purification and use of stem cells from cord blood for research purposes is permitted by an Italian law after obtaining informed consent from the mothers (art. 2, paragraph 1, letter f, decree of 18 November 2009). ECFCs were grown in EGM-2 culture medium (Lonza, Lonn, Swiss), supplemented with 10% FBS (Euroclone, Milan, Italy) onto gelatin-coated dishes. ECFCs were grown in a humified atmosphere with 5% of CO_2_ in air and the medium was refreshed every 2 days.

### 4.2. Co-Cultures

HUVECs (6 × 10^4^) were seeded into gelatin-coated 6-well plates. The next day, A375 was seeded onto membrane inserts (Millicell Cell Culture Plate Insert, 30 mm, PCF Polypropylene carb, 0.4 µm, Merck Millipore, Milan, Italy) and moved to the 6-well plate on which the HUVECs were seeded earlier. The two cell types shared the culture medium but were separated by the membrane of the insert. The medium used was DMEM without any growth factor and supplemented with 5% FCS. Cells were co-cultured for 6–9 and 24 h. Then, supernatants were collected for ELISA assay and for gelatin zymography.

### 4.3. Real-Time PCR

Total RNA was isolated using TRI Reagent and spectroscopically quantified by NanoDrop (ThermoFisher Scientific, Milan, Italy) instrument. One μg of total RNA was reverse transcribed by means of Prime Script RT reagent Kit with gDNA eraser (Takara, Otsu, Japan), and cDNA was amplified with specific primers described in Cecchi et al. [7]. Quantitative real-time PCR (qRT-PCR) was performed by SYBR Premix Ex Taq kit (Takara, Otsu, Japan) according to the manufacturer’s instructions using a Rotorgene RG-3000A system (Qiagen, Monza, Italy). 18s rRNA was confirmed to be stable and was used as the internal control. qRT-PCR was performed using the following procedure: 98 °C for 2 min, 40 cycles of 98 °C for 5 s, 60 °C for 10 s. The program was set to reveal the melting curve of each amplicon from 60 to 95 °C with a read every 0.5 °C. Data analysis was performed by CT method (Rotore-Gene 6000 Series software 1.7).

### 4.4. Sanger Sequencing

cDNA samples obtained from HUVECs and ECFCs (pre- and post-real-time amplification) were directly sequenced, in both directions, using the same primers used for real-time PCR with the BigDye™ Terminator v3.1–Cycle Sequencing Kit (Thermo Fisher Scientific, Milan, Italy). Sequences obtained using 3500 Dx Genetic Analyzer (CE-IVD, IVDR) were analyzed and compared with the reported TDO2 gene structure (ENST00000536354.3, NM_005651.4) using the dedicated software (SeqScape™ Software v4.0).

### 4.5. Immunofluorescence

HUVECs and ECFCs were seeded (30 × 10^4^ cells) in Endothelial Basal medium with 10% FBS into ibidi slides chamber (ibidi GmbHcoated, Gräfelfing, Germany) and incubated in a 5% CO_2_ atmosphere at 37 °C for 24 h. After fixation with cold methanol for 5 min, immunofluorescence analysis was performed on cells. The blocking of non-specific binding sites was performed with 10 mg/mL bovine serum albumin with 0.2% triton X-100 (Sigma-Aldrich, Milan, Italy) added in PBS for 1 h at room temperature. Then, the following primary antibodies were added for overnight incubation at 4 °C: the monoclonal mouse anti human-TDO (1:200; Novus Biologicals, Briarwood Ave, OH, USA) or the polyclonal rabbit anti-human IDO1 (1:200; Abcam, Cambridge, UK) or the monoclonal rabbit anti-human AhR (1:100; Cell Signaling Technology, Danvers, MA, USA). Cells which were not incubated with primary antibodies were used as negative controls. The next morning, after multiple washes, cells were incubated for 2 h at room temperature with secondary goat anti-rabbit or anti-mouse antibodies, conjugated with Goat anti-Rabbit IgG secondary antibody AlexaFluor488 (green fluorescence) or Goat anti-Mouse IgG secondary antibody AlexaFluor555 (red-orange fluorescence) (all from ThermoFisher Scientific, Milan, Italy). Green signal was amplified using the anti-FITC Fluorescein/Oregon green antibody for 1 h 30 min (1:100; ThermoFisher Scientific, Milan, Italy) at room temperature. Nuclei were labelled with Hoechst 33342 (20 μg/mL; Sigma-Aldrich; blue fluorescence). Slides were then washed with PBS and examined with Leica SP8 microscope digital color camera and Leica DC Viewer software. Cell fluorescence intensity was measured using ImageJ. Briefly, cells were selected and then the area integrated intensity and mean grey value were measured. Then, the Corrected Total Cell Fluorescence (CTCF) was calculated using the following formula:

CTCF = Integrated Density − (Area of selected cell × Mean fluorescence of background readings)

In order to assess AhR nuclear translocation, Mander’s coefficient (M1) was determined with ImageJ 1.54d software. 

### 4.6. Cell Proliferation

Cell proliferation was assessed by MTT (3-(4,5-dimethylthiazol-2-yl)-2,5-diphenyltetrazoliumbromid) assay. Briefly, cells (1.5 × 10^3^/100 μL) were plated on flat-bottom 96-multiwell plates and allowed to adhere overnight. Starving condition (1% FCS) for 24 h was applied to cells that were then pretreated for 1 h with 680C91 (40 µM) or epacadostat (1 µM) and stimulated with 10 ng/mL FGF-2 or 20 ng/mL VEGF-A. After 48 h MTT (final concentration 5 mg/mL) was added to each well, followed by 4 h of incubation at 37 °C. Formazan crystals were dissolved in 100 μL Isopropanol, per well, followed by recording the absorbance at 570 nm by a microplate reader (Victor Nivo 2F, Perkin Elmer, Milan, Italy).

### 4.7. In Vitro Capillary Morphogenesis

In vitro capillary morphogenesis was performed in tissue culture wells coated with Geltrex™ matrix (ThermoFisher Scientific, Milan, Italy) as described [20]. ECFCs were plated (18 × 10^3^/well) in EGM-2 medium without growth factors, supplemented with 2% FBS and incubated at 37 °C-5% CO_2_. Pictures were acquired at regular intervals with an EVOS optical microscope (Thermo Fisher Scientific, Monza, Italy). The results were quantified at 6 h using the Angiogenesis Analyzer tool of ImageJ software 19 that was used to perform the statistical analysis for each experimental condition. “Nodes” are identified as pixels that have at least three neighbors, corresponding to a bifurcation. “Junctions” are elements composed of several nodes. “Segments” are elements delimited by two junctions. “Meshes” are the polygon structures reinforced with more than one layer of cells in their walls and have also been referred to by other authors as a ‘Honeycomb formation’. Six to nine photographic fields from three plates were scanned for each point. 

### 4.8. ELISA Assay for Kynurenine Determination

Cell-conditioned media were collected, clarified by centrifugation at 14,000 rpm for 10 min and supernatants were stored at −80 °C. Kyn concentration was measured by ELISA immunoassay (ImmuSmol, Bordeaux, France) according to the manufacturer’s instructions.

### 4.9. Western Blot Analysis

After harvesting the cells, samples were resuspended in 20 mM RIPA buffer (pH 7.4) (Merk Millipore, Vimodrone, MI, Italy), which contains a mixture of proteinase inhibitors (Calbiochem, Merck, Darmstadt, Germany), and treated by sonication (Microson XL-2000, Minisonix, Farmingdale, NY, USA). Then, the samples were centrifugated at 14,000 rpm for 7 min at 4 °C to get rid of cellular debris, and the supernatants were collected. An amount of supernatants containing equal concentration of protein (30 μg) in Laemmli buffer was separated on Bolt^®^ Bis-Tris Plus gels 4–12% precast polyacrylamide gels (Life Technologies, Monza, Italy) and transferred as reported [47]. Blots were blocked for 1 h, at room temperature, with 5% milk in PBS 0.1% tween solution. Then, the membranes were incubated at 4 °C overnight with the following primary antibodiesrabbit anti-pmTOR (Ser2448) (1:1000 Cell signaling Technology, Cat#2971), rabbit anti-mTOR (1:1000 Abcam Cat# ab2732), rabbit anti-cMyc (1:1000, Abcam Cat# ab32072), rabbit anti-Klf4 (1:1000, St Cruz Biotechnology, Dallas, TX, USA, Cat# sc-20691), rabbit GAPDH antibody (1:1000, Cell signaling Technology, Cat# 2118) or mouse monoclonal GAPDH antibody (1:5000, Merck Cat# G8795). GAPDH was used to assess an equal amount of protein loaded in each lane. Anti-rabbit IgG (whole molecule)–Peroxidase antibody (Sigma, Cat#A0545) or anti-mouse IgG (whole molecule)–Peroxidase antibody (Sigma, Cat#A9044) were used as secondary antibodies; the enhanced chemiluminescence (ECL) procedure was employed for development.

### 4.10. Gelatin Zymography

Following the co-culture experiments, the media were harvested, clarified by centrifugation and loaded onto 8% SDS-PAGE containing 1 mg/mL gelatin under non-denaturing conditions to perform electrophoresis. After protein separation, the gels were washed with 2.5% Triton X-100 to remove SDS and incubated for 24 h at 37 °C in 50 mM Tris buffer containing 200 mM NaCl and 20 mM CaCl_2_, pH 7.4. The gels were then stained with 0.1% Coomassie brilliant blue R-250 in 10% acetic acid and 45% methanol and destained with 10% acetic acid and 45% methanol. The areas of gelatinase activity appeared transparent against a blue background. Gelatinase activity was then evaluated by quantitative densitometry (Image J 1.54d software).

### 4.11. Statistical Analysis

Statistical analysis was performed using GraphPad Prism 8.00 (GraphPad Software, San Diego, CA, USA). Parametric data were reported as means ± SEM and differences between groups were tested with ANOVA test (followed by Bonferroni’s and Dunnett’s Multiple Comparison Test). Nonparametric data were analyzed using the Kruskal–Wallis test, followed by Dunn’s post hoc test. For comparisons between two groups, we used Student’s unpaired *t* test. Alpha value was set at 0.05.

## Data Availability

Data are available at the Department of Health Sciences, University of Florence, Viale Pieraccini 6, 50139, Florence, Italy.

## Supplementary Materials

The following supporting information can be downloaded at: https://www.mdpi.com/article/10.3390/ph17050558/s1, Figure S1: TDO2 Sequencing; Figure S2: RT-PCR for IDO1 expression in A375, HUVECs and co-cultures.

## References

[1] Badawy A.A.B. (2017). Kynurenine Pathway of Tryptophan Metabolism: Regulatory and Functional Aspects. Int. J. Tryptophan Res..

[2] Schramme F., Crosignani S., Frederix K., Hoffmann D., Pilotte L., Stroobant V., Preillon J., Driessens G., van den Eynde B.J. (2020). Inhibition of Tryptophan-Dioxygenase Activity Increases the Antitumor Efficacy of Immune Checkpoint Inhibitors. Cancer Immunol. Res..

[3] Long G.V., Dummer R., Hamid O., Gajewski T.F., Caglevic C., Dalle S., Arance A., Carlino M.S., Grob J.J., Kim T.M. (2019). Epacadostat plus Pembrolizumab versus Placebo plus Pembrolizumab in Patients with Unresectable or Metastatic Melanoma (ECHO-301/KEYNOTE-252): A Phase 3, Randomised, Double-Blind Study. Lancet Oncol..

[4] Oweira H., Lahdou I., Mehrle S., Khajeh E., Nikbakhsh R., Ghamarnejad O., Terness P., Reißfelder C., Sadeghi M., Ramouz A. (2023). Kynurenine Is the Main Metabolite of Tryptophan Degradation by Tryptophan 2,3-Dioxygenase in HepaRG Tumor Cells. J. Clin. Med..

[5] Zhong C., Peng L., Tao B., Yin S., Lyu L., Ding H., Yang X. (2022). TDO2 and Tryptophan Metabolites Promote Kynurenine/AhR Signals to Facilitate Glioma Progression and Immunosuppression. Am. J. Cancer Res..

[6] Cecchi M., Paccosi S., Silvano A., Eid A.H., Parenti A. (2021). Dexamethasone Induces the Expression and Function of Tryptophan-2-3-Dioxygenase in SK-MEL-28 Melanoma Cells. Pharmaceuticals.

[7] Cecchi M., Mannini A., Lapucci A., Silvano A., Lulli M., Luceri C., D’Ambrosio M., Chiarugi A., Eid A.H., Parenti A. (2022). Dexamethasone Promotes a Stem-Like Phenotype in Human Melanoma Cells via Tryptophan 2,3 Dioxygenase. Front. Pharmacol..

[8] Dewing D., Emmett M., Pritchard Jones R. (2012). The Roles of Angiogenesis in Malignant Melanoma: Trends in Basic Science Research over the Last 100 Years. ISRN Oncol..

[9] Pandita A., Ekstrand M., Bjursten S., Zhao Z., Fogelstrand P., Le Gal K., Ny L., Bergo M.O., Karlsson J., Nilsson J.A. (2021). Intussusceptive Angiogenesis in Human Metastatic Malignant Melanoma. Am. J. Pathol..

[10] Dome B., Timar J., Ladanyi A., Paku S., Renyi-Vamos F., Klepetko W., Lang G., Dome P., Bogos K., Tovari J. (2009). Circulating Endothelial Cells, Bone Marrow-Derived Endothelial Progenitor Cells and Proangiogenic Hematopoietic Cells in Cancer: From Biology to Therapy. Crit. Rev. Oncol. Hematol..

[11] Tasev D., Koolwijk P., Van Hinsbergh V.W.M. (2016). Therapeutic Potential of Human-Derived Endothelial Colony-Forming Cells in Animal Models. Tissue Eng. Part B Rev..

[12] Fujisawa T., Tura-Ceide O., Hunter A., Mitchell A., Vesey A., Medine C., Gallogly S., Hadoke P.W.F., Keith C., Sproul A. (2019). Endothelial Progenitor Cells Do Not Originate From the Bone Marrow. Circulation.

[13] Biagioni A., Laurenzana A., Menicacci B., Peppicelli S., Andreucci E., Bianchini F., Guasti D., Paoli P., Serratì S., Mocali A. (2021). UPAR-Expressing Melanoma Exosomes Promote Angiogenesis by VE-Cadherin, EGFR and UPAR Overexpression and Rise of ERK1,2 Signaling in Endothelial Cells. Cell. Mol. Life Sci..

[14] Laurenzana A., Biagioni A., D’Alessio S., Bianchini F., Chillà A., Margheri F., Luciani C., Mazzanti B., Pimpinelli N., Torre E. (2014). Melanoma Cell Therapy: Endothelial Progenitor Cells as Shuttle of the MMP12 UPAR-Degrading Enzyme. Oncotarget.

[15] Pan J., Yuan K., Shanshan P., Yanqin H., Yujuan Z., Hu Y., Yuanyuan F., Shi Y., Liu Y., Hongmei W. (2017). Gene Silencing of Indoleamine 2,3-Dioxygenase Hinders Tumor Growth through Angiogenesis Inhibition. Int. J. Oncol..

[16] Zhang W., Mao S., Shi D., Zhang J., Zhang Z., Guo Y., Wu Y., Wang R., Wang L., Huang Y. (2019). MicroRNA-153 Decreases Tryptophan Catabolism and Inhibits Angiogenesis in Bladder Cancer by Targeting Indoleamine 2,3-Dioxygenase 1. Front. Oncol..

[17] Wei L., Zhu S., Li M., Li F., Wei F., Liu J., Ren X. (2018). High Indoleamine 2,3-Dioxygenase Is Correlated With Microvessel Density and Worse Prognosis in Breast Cancer. Front. Immunol..

[18] Hoffmann D., Dvorakova T., Stroobant V., Bouzin C., Daumerie A., Solvay M., Klaessens S., Letellier M.C., Renauld J.C., van Baren N. (2020). Tryptophan 2,3-Dioxygenase Expression Identified in Human Hepatocellular Carcinoma Cells and in Intratumoral Pericytes of Most Cancers. Cancer Immunol. Res..

[19] Paccosi S., Cecchi M., Silvano A., Fabbri S., Parenti A. (2020). Different Effects of Tryptophan 2,3-Dioxygenase Inhibition on SK-Mel-28 and HCT-8 Cancer Cell Lines. J. Cancer Res. Clin. Oncol..

[20] Margheri F., Chillà A., Laurenzana A., Serratì S., Mazzanti B., Saccardi R., Santosuosso M., Danza G., Sturli N., Rosati F. (2011). Endothelial Progenitor Cell-Dependent Angiogenesis Requires Localization of the Full-Length Form of UPAR in Caveolae. Blood.

[21] Li T., Fu J., Zeng Z., Cohen D., Li J., Chen Q., Li B., Liu X.S. (2020). TIMER2.0 for Analysis of Tumor-Infiltrating Immune Cells. Nucleic Acids Res..

[22] Rossi S., Cordella M., Tabolacci C., Nassa G., D’Arcangelo D., Senatore C., Pagnotto P., Magliozzi R., Salvati A., Weisz A. (2018). TNF-Alpha and Metalloproteases as Key Players in Melanoma Cells Aggressiveness. J. Exp. Clin. Cancer Res..

[23] Goździalska A., Wojas-Pelc A., Drąg J., Brzewski P., Jaśkiewicz J., Pastuszczak M. (2016). Expression of Metalloproteinases (MMP-2 and MMP-9) in Basal-Cell Carcinoma. Mol. Biol. Rep..

[24] Deryugina E.I., Quigley J.P. (2015). Tumor Angiogenesis: MMP-Mediated Induction of Intravasation- and Metastasis-Sustaining Neovasculature. Matrix Biol..

[25] Eddy K., Chen S. (2020). Overcoming Immune Evasion in Melanoma. Int. J. Mol. Sci..

[26] Filippi L., Bruno G., Domazetovic V., Favre C., Calvani M. (2020). Current Therapies and New Targets to Fight Melanoma: A Promising Role for the Β3-Adrenoreceptor. Cancers.

[27] Ramelyte E., Schindler S.A., Dummer R. (2017). The Safety of Anti PD-1 Therapeutics for the Treatment of Melanoma. Expert Opin. Drug Saf..

[28] Liu Q., Nie R., Li M., Li L., Zhou H., Lu H., Wang X. (2021). Identification of Subtypes Correlated with Tumor Immunity and Immunotherapy in Cutaneous Melanoma. Comput. Struct. Biotechnol. J..

[29] Folkman J., Merler E., Abernathy C., Williams G. (1971). Isolation of a Tumor Factor Responsible for Angiogenesis. J. Exp. Med..

[30] Haibe Y., Kreidieh M., El Hajj H., Khalifeh I., Mukherji D., Temraz S., Shamseddine A. (2020). Resistance Mechanisms to Anti-Angiogenic Therapies in Cancer. Front. Oncol..

[31] Huang M., Lin Y., Wang C., Deng L., Chen M., Assaraf Y.G., Chen Z.S., Ye W., Zhang D. (2022). New Insights into Antiangiogenic Therapy Resistance in Cancer: Mechanisms and Therapeutic Aspects. Drug Resist. Updat..

[32] Vasudev N.S., Reynolds A.R. (2014). Anti-Angiogenic Therapy for Cancer: Current Progress, Unresolved Questions and Future Directions. Angiogenesis.

[33] Nonaka H., Saga Y., Fujiwara H., Akimoto H., Yamada A., Kagawa S., Takei Y., Machida S., Takikawa O., Suzuki M. (2011). Indoleamine 2,3-Dioxygenase Promotes Peritoneal Dissemination of Ovarian Cancer through Inhibition of Natural Killercell Function and Angiogenesis Promotion. Int. J. Oncol..

[34] Brouillet S., Hoffmann P., Benharouga M., Salomon A., Schaal J.P., Feige J.J., Alfaidy N. (2010). Molecular Characterization of EG-VEGF-Mediated Angiogenesis: Differential Effects on Microvascular and Macrovascular Endothelial Cells. Mol. Biol. Cell.

[35] Zabroski I.O., Nugent M.A. (2021). Lipid Raft Association Stabilizes VEGF Receptor 2 in Endothelial Cells. Int. J. Mol. Sci..

[36] Folgiero V., Miele E., Carai A., Ferretti E., Alfano V., Po A., Bertaina V., Goffredo B.M., Benedetti M.C., Camassei F.D. (2016). IDO1 Involvement in MTOR Pathway: A Molecular Mechanism of Resistance to MTOR Targeting in Medulloblastoma. Oncotarget.

[37] Barati M., Akhondi M., Mousavi N.S., Haghparast N., Ghodsi A., Baharvand H., Ebrahimi M., Hassani S.N. (2021). Pluripotent Stem Cells: Cancer Study, Therapy, and Vaccination. Stem Cell Rev. Rep..

[38] Alvarado A.G., Tessema K., Muthukrishnan S.D., Sober M., Kawaguchi R., Laks D.R., Bhaduri A., Swarup V., Nathanson D.A., Geschwind D.H. (2022). Pathway-Based Approach Reveals Differential Sensitivity to E2F1 Inhibition in Glioblastoma. Cancer Res. Commun..

[39] Alla V., Engelmann D., Niemetz A., Pahnke J., Schmidt A., Kunz M., Emmrich S., Steder M., Koczan D., Pützer B.M. (2010). E2F1 in Melanoma Progression and Metastasis. J. Natl. Cancer Inst..

[40] Ray S.K. (2019). Emerging Evidence for Krüppel-Like Factor 4 (KLF4) as a Tumor Suppressor in Neuroblastoma. Neuroblastoma.

[41] He Z., He J., Xie K. (2023). KLF4 Transcription Factor in Tumorigenesis. Cell Death Discov..

[42] Kim J., Liu Y., Qiu M., Xu Y. (2016). Pluripotency Factor Nanog Is Tumorigenic by Deregulating DNA Damage Response in Somatic Cells. Oncogene.

[43] Clemente-Periván S.I., Gómez-Gómez Y., Leyva-Vázquez M.A., Lagunas-Martínez A., Organista-Nava J., Illades-Aguiar B. (2020). Role of Oct3/4 in Cervical Cancer Tumorigenesis. Front. Oncol..

[44] Shen L., Qin K., Wang D., Zhang Y., Bai N., Yang S., Luo Y., Xiang R., Tan X. (2014). Overexpression of Oct4 Suppresses the Metastatic Potential of Breast Cancer Cells via Rnd1 Downregulation. Biochim. Biophys. Acta.

[45] Zhang X., Zhang Y., Jia Y., Qin T., Zhang C., Li Y., Huang C., Liu Z., Wang J., Li K. (2020). Bevacizumab Promotes Active Biological Behaviors of Human Umbilical Vein Endothelial Cells by Activating TGFβ1 Pathways via Off-VEGF Signaling. Cancer Biol. Med..

[46] Corselli M., Parodi A., Mogni M., Sessarego N., Kunkl A., Dagna-Bricarelli F., Ibatici A., Pozzi S., Bacigalupo A., Frassoni F. (2008). Clinical Scale Ex Vivo Expansion of Cord Blood-Derived Outgrowth Endothelial Progenitor Cells Is Associated with High Incidence of Karyotype Aberrations. Exp. Hematol..

[47] Laurenzana A., Margheri F., Biagioni A., Chillà A., Pimpinelli N., Ruzzolini J., Peppicelli S., Andreucci E., Calorini L., Serratì S. (2019). EGFR/UPAR Interaction as Druggable Target to Overcome Vemurafenib Acquired Resistance in Melanoma Cells. eBioMedicine.

