# Supplementary material for: Unveiling the Role of Tryptophan 2,3-Dioxygenase in the Angiogenic Process
Source: Pharmaceuticals (Basel). 2024 Apr 27;17(5):558. doi: 10.3390/ph17050558 (PMC11124529; doi:10.3390/ph17050558)
Supplement: Supplementary file 1 [file pharmaceuticals-17-00558-s001.zip › pharmaceuticals-2864793-supplementary.pdf]

A

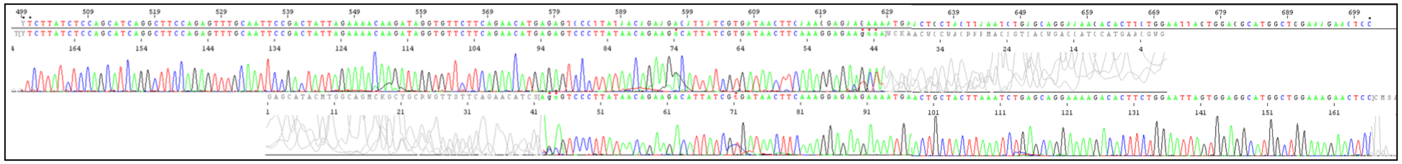

B

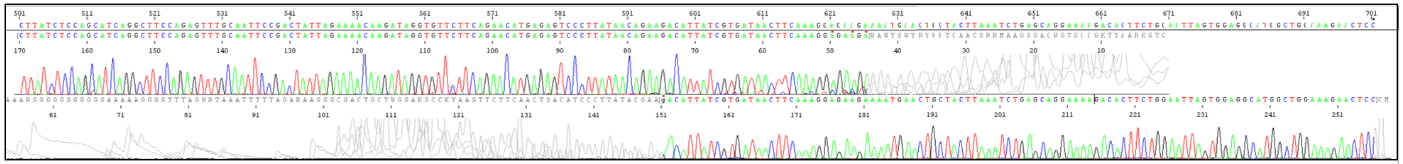

Supplementary Figure S1. **TDO2 Sequencing**: A) sequence related to HUVEC cDNA that shows end of exon 6 and exon 7 of TDO2 amplified region of transcript. B) sequence related to ECFC cDNA that shows an amplified region spanning between exon 6 and exon 7 of TDO2 transcript as in the HUVEC.

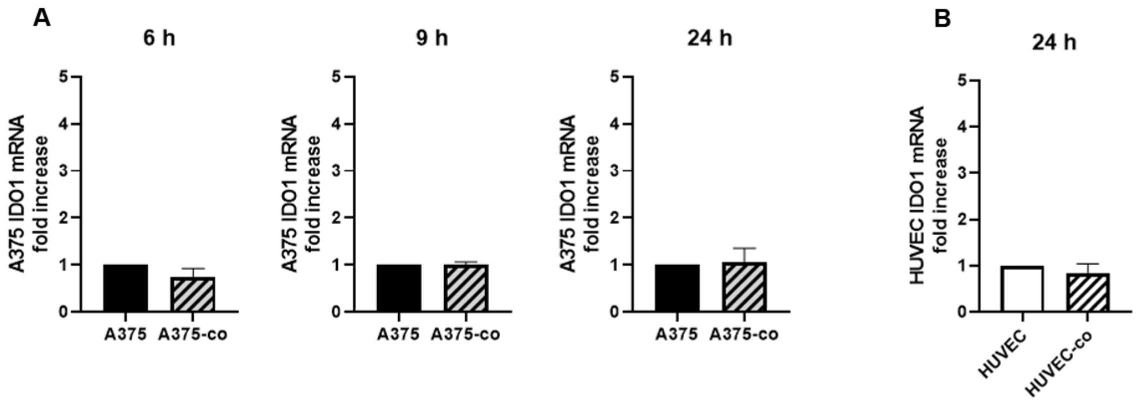

Supplementary Figure S2. A, B) RT-PCR for IDO1 expression in A375 , HUVECs and co-cultures. A) RT-PCR in A375 alone and in A375 co-cultured with HUVECs (A375-co). Time-course. B) RT-PCR in HUVECs alone and in HUVECs co-cultured with A375 (HUVEC-co). Mean ± SEM, n=3.
